# Hydropneumothorax With Bronchopleural Fistula Following the Activation of Mycobacterium tuberculosis: A Case Report

**DOI:** 10.7759/cureus.40844

**Published:** 2023-06-23

**Authors:** Maitha Al-Neyadi, Salma Alghfeli, Mustak Dukandar

**Affiliations:** 1 Emergency Department, Tawam Hospital, Al Ain, ARE

**Keywords:** video-assisted thoracoscopic surgery (vats), persistent air leak (pal), mycobacterium tuberculosis, hydropneumothorax, diabetes mellites, bronchopleural fistula

## Abstract

Tuberculosis is not a disease of the current era; failure to eradicate it continues to cause unusual complications, which results in detrimental sequelae to the patients. It usually presents with respiratory symptoms such as shortness of breath, cough, and fever, in addition to extrapulmonary symptoms. While there have been a few published case reports on patients presenting with hydropneumothorax due to tuberculosis, its occurrence is relatively rare. Furthermore, to the best of our knowledge, this is the first published case of hydropneumothorax due to tuberculosis within the United Arab Emirates, as confirmed by a search on PubMed. Here, we present a case of a young farmer from Bangladesh who presented with shortness of breath and fever and was found to have decreased air entry along with hyperresonance sounds on examination. Fortunately, the patient was in a stable state, required minimum oxygen therapy, and was not escalated for further noninvasive or invasive mechanical ventilation. The patient was admitted to a tertiary hospital to receive initial medical therapy interim to transfer the patient to a facility where thoracic surgeons are found.

## Introduction

Tuberculosis is caused by gram-negative bacteria, namely, *Mycobacterium tuberculosis*. It resides in the lungs most of the time, making it the most common organ involved in that disease. Still, other organs are equally involved such as the lymphoreticular, gastrointestinal and hepatic, cutaneous, central nervous, musculoskeletal, and reproductive systems [1]. *Mycobacterium tuberculosis* continues to have peculiar presentations, specifically in the young generation, followed by unpredictable complications. Hydropneumothorax due to tuberculosis is an unusual presentation, although it is not uncommon to present with other pathogens. Few case reports have been published on patients presenting with hydropneumothorax and tuberculosis. Hydropneumothorax occurs when an abscess forms that ruptures eventually or during an infective process or even when the thoracic duct is disrupted due to blockage [2]. It is diagnosed using a plain erect chest radiograph that showed the gas fluid level. Computed tomography (CT) was performed to confirm the diagnosis and rule out bronchopulmonary fistula. The objective of this case report is to present one of the rare tuberculosis complications associated with a finding that adds up to the morbidity following reactivation in a young diabetic immigrant.

## Case presentation

A 38-year-old male farmer from Bangladesh with uncontrolled diabetes who was on oral hypoglycemic agents presented to the emergency department (ED) with worsening shortness of breath over a period of three months. He deteriorated over the last five days from presentation, where he primarily had worsened shortness of breath associated with productive cough and intermittent fever. The patient denied chest pain, weight loss, night sweats, or headache. There was no abdominal pain, and he had normal bowel movements and urination. The patient never experienced similar symptoms in the past.

The patient was initially admitted to a remote hospital, and primary measures and basic support were provided. His vital signs were as follows: temperature of 39.6°C, heart rate of 131 beats per minute, blood pressure of 144/97 mmHg, and oxygen saturation of 94% at its lowest, placed on 2 L nasal cannula, and 98% at its peak. His vital signs reflected having mild respiratory distress. Initial management included 1 g of paracetamol, and the fever settled, and broad-spectrum antibiotics were given as pneumonia was suspected.

**Figure 1:**
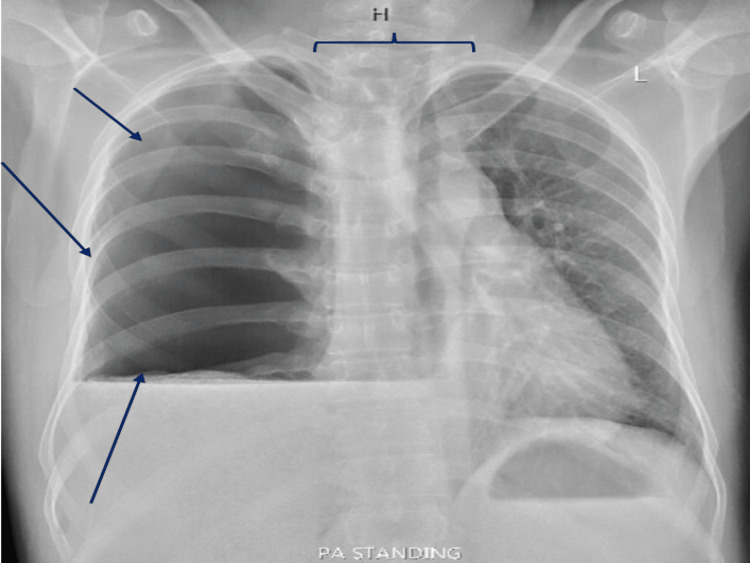
The image reveals a large air-fluid level in the right chest, suggestive of hydropneumothorax (blue arrows). There is a leftward shift of the mediastinum (horizontal bracket), while the left lung appears normal. The heart and aorta are unremarkable, showing no signs of abnormalities. The bones show no obvious fracture.

Chest radiography revealed a large hydropneumothorax on the right side and a mediastinal shift to the left side; the rest of the report was unremarkable (Figure 1). Blood tests were conducted and sent for basic laboratory tests, inflammatory markers, and a coagulation panel. The patient was transferred in a stable state to a tertiary care hospital for further evaluation and monitoring.

**Figure 2:**
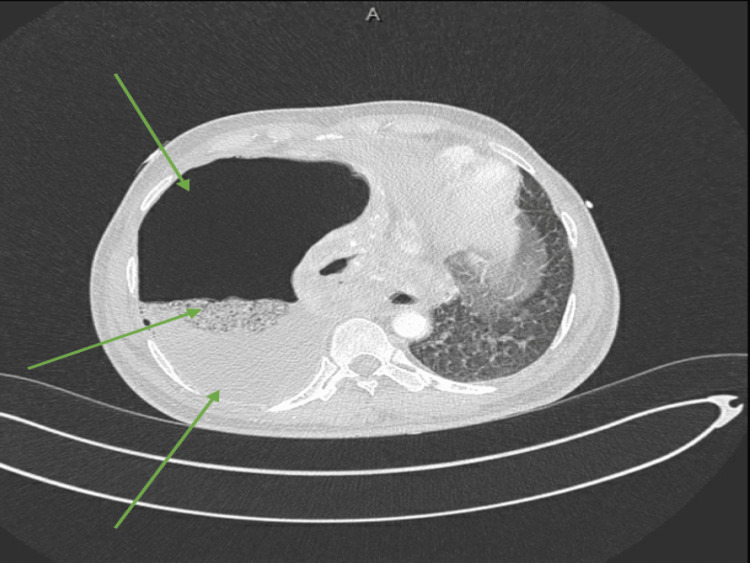
A substantial air-fluid cavity is evident in the right hemithorax, strongly suggestive of an empyema (bottom arrow) causing the collapse of the right lung (middle arrow). The lower lobe shows breakdown, raising the possibility of communication with the empyema and suggesting the potential presence of a bronchopleural fistula. However, definitive confirmation cannot be ruled out at this stage. These findings suggest the likelihood of chronic infection.

Upon arrival at the emergency department (ED), the patient was alert, fully conscious, and oriented, scoring a Glasgow Coma Scale (GCS) of 15/15. However, the patient presented with tachypnea; the rest of the vital signs were within normal limits. Physical examination revealed decreased air entry on the right side of the chest. Trachea was midline. The rest of the systemic examinations were unremarkable. Additional investigations in the ED were added, including a computed tomography (CT) scan, acid-fast bacilli (AFB) smear, serological testing, and other basic baseline functions such as hepatic panel (Figure 2).

Consultations were made with thoracic surgery, pulmonology, and interventional radiology. The consensus between thoracic surgery and interventional radiology was to refrain from inserting a chest tube at the current time and instead opt for surgical drainage. This is attributed to a possible communication between the bronchial tree and the pleural cavity resulting in persistent leak from the destructed and infected lung. On the other hand, pulmonology advised treating the infective cause and considering fluid drainage.

Elevated inflammatory markers were observed as anticipated, and hyponatremia was noticed in conjunction with elevated glucose levels. Subsequently, the patient was admitted to the medical floor with appropriate precautions in place, including contact, droplet, and airborne precautions.

The patient was hospitalized for four days, during which he was closely monitored and administered intravenous antibiotics. A pigtail was inserted to the right chest as the patient was in pain and dyspneic. The fluid is purulent; the patient is less dyspneic, and pain is controlled. A sample of the drained fluid was sent to the laboratory for analysis, revealing a pH level of 6.5, lactate dehydrogenase (LDH) level of 995, and a protein level of 52. Additional results can be found in Table 1. A chest X-ray was added post-pigtail insertion.

**Table 1:** Pleural fluid analysis. LDH: lactate dehydrogenase

Parameter	Result	Normal Value
Appearance	Milky	Pale yellow
Color	Pale yellow	Straw-colored
RBC	152,000×10^6^	None
Nucleated cells	358,351×10^6^	None
Pathology	Mostly degenerated cells	No abnormal cells
Cholesterol	1.40 mmol/L	Parallel to serum value
Protein	54 g/L	1-2 g/dL
Glucose	14.9 mmol/L	Parallel to serum value
LDH	37,483 IU/L	Parallel to serum value
pH	6.50	7.37-7.43
Polymorphous cells (%)	80.0%	None
Monomorphous cells (%)	19.2%	None
Body fluid culture	Enterobacter hormaechei	No growth

**Figure 3:**
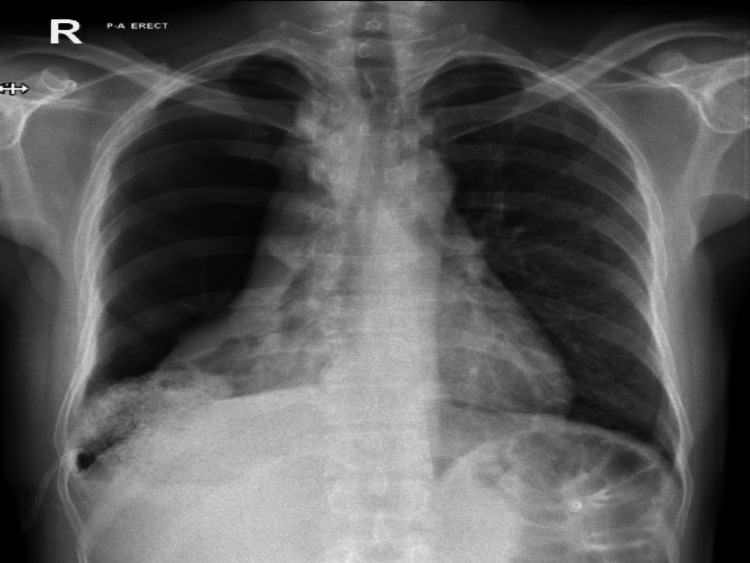
Right-sided pigtail drain in situ. Interval reduction in right-sided pneumothorax. Collapsed right lung. Normal heart size. The left lung is clear.

Cytology and histopathology were performed for our patient. Pleural fluid cytology showed heavy inflammatory infiltrate, predominantly of neutrophils, while histology concluded extensive necrotizing and suppurative inflammation with Langerhans multinucleated giant cells, with abundant fibrinopurulent exudate. It is highly suggestive of an infective pleurisy suggesting tuberculosis (Figure 3).

The final acid-fast bacilli (AFB) results were consistently positive on three separate occasions.

The patient was transferred to a facility where thoracic surgeons were available. The patient was evaluated by the team and underwent right thoracoscopic pleural washout, partial decortication, rib resection, and drainage. This procedure was followed with chest tube insertion. As for the bronchopleural fistula (BPF), nothing in specific was done during the surgery. The patient was planned to be observed postoperatively for persistent air leak. In case he did, the patient will undergo upper and middle lobe decortication and lower lobectomy.

The procedure was uneventful, and he was extubated postoperatively and transferred to the intensive care unit (ICU). He stayed in the ICU for a total of two days and then stepped down to the medical floor. Infectious diseases were on board for the initiation of antituberculosis medications. They started him on rifampicin, isoniazid, pyridoxine, pyrazinamide, and ethambutol, and topped with multivitamins and B complex.

**Figure 4:**
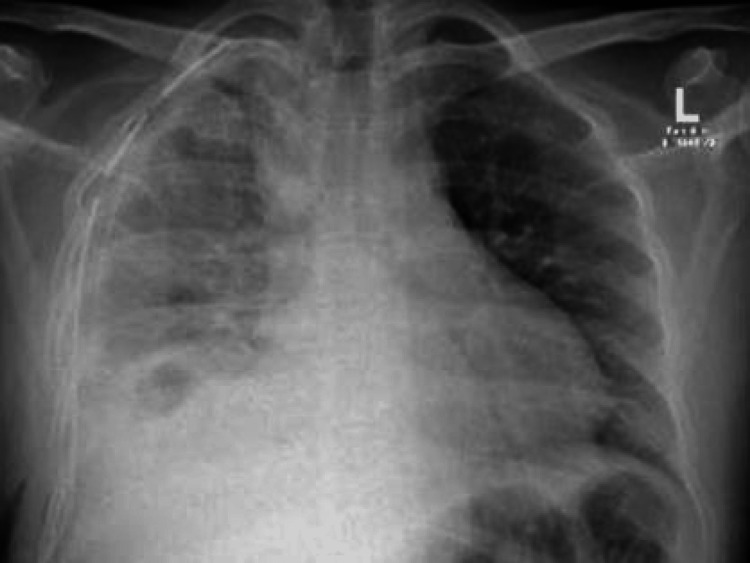
Right-sided chest drain with the tip projected at the right apex. There is almost complete resolution of right-sided pneumothorax with the reexpansion of the right lung. Ground-glass opacification was suggestive of right-sided pleural effusion with basal atelectasis.

The postoperative chest radiograph is presented in Figure 4.

On the medical floor, the patient was monitored throughout; frequent and then intermittent chest X-rays were done. He was treated for electrolyte imbalances and was optimized medically for diabetic control, pain management, drain care, ambulation, and diet control. The patient remained in stable condition; antituberculosis medications and appropriate antibiotics were administered; he did not deteriorate at any stage. He was discharged after 23 days of hospital stay to an infection-dedicated hospital.

## Discussion

*Mycobacterium tuberculosis* is a significant global health burden, infecting 10 million individuals worldwide and resulting in 1.5 million deaths. It is ranked as the 13th leading cause of death globally and is considered the second most infectious cause [3]. It includes pulmonary and extrapulmonary signs and symptoms, from shortness of breath, cough, and fever to weight loss and night sweats, varying among individuals.

*Mycoplasma pneumoniae* behaves destructively when residing in the lungs, causing one of the devastating complications of pneumonia, specifically necrotizing pneumonia with pleural effusion, and resulting in the destruction of the lung parenchyma, vessels, and arteries, leading to hydropneumothorax with respiratory distress and failure [4]. In reference to a case report in Korea in 2016, they have concluded that those having *Mycobacterium avium* complex (MAC) infection with pleural effusion and the presence of bronchopleural fistula on CT point toward MAC infection as the cause of effusion [5].

Latent tuberculosis activation occurs in around 5%-15% of infected individuals within the first five years of acquiring the infection [6]. Reactivation depends on the host's immune response to the presence of diabetes. Susceptibility to activation depends on the expression of cytokines [7].

Hydropneumothorax is the presence of both air and fluid in the pleural cavity, occurring as a result of infective or noninfective causes. While hydropneumothorax has been extensively studied, the coexistence of hydropneumothorax with tuberculosis is often underreported in the literature. However, tuberculosis is considered the most common culprit of hydropneumothorax [8]. In tuberculosis, the condition typically starts with a focus of infection, which can progress to form a cavity that eventually ruptures, releasing air and fluid into the pleural cavity. Moreover, it is the disequilibrium between the production and absorption of air and fluid in the pleural cavity [9]. With a negative intrapleural cavity pressure and the presence of disequilibrium, damage continues to occur, leading to the deterioration of the clinical picture [10].

Hydropneumothorax, a subtype of pneumothorax, typically presents with sudden dyspnea and unilateral chest pain associated with desaturation, tachycardia, and tachypnea. Physical examination reveals decreased air entry that is followed by obliterated lungs on chest X-ray. A CT scan is done to evaluate the consistency of the fluid and, more importantly, the presence of a bronchopleural fistula [11]. This is better followed with fluid analysis to identify the causative agent in order to initiate tailored therapy.

Chest tube insertion may be considered a definitive treatment or an interim option to the surgical procedure. There is no harm in inserting it; indeed, fluid should be drained as it helps relieve the patient's symptoms and guide therapy. As stated above, hydropneumothorax is associated with tuberculosis. Therefore, it is essential to adhere to standard precautionary measures for airborne infections, including the use of at least an N95 respirator, to ensure the safety of healthcare providers and minimize the risk of transmission.

On the contrary, the patient might be placed at risk of developing tension pneumothorax from misplacement or clogged chest tube. That might also be complicated when bronchopleural fistula (BPF) is present [8], namely, linked to high morbidity and longer hospital stay. The presence of persistent air leak raises high suspicion of bronchopleural fistula [12], after intercostal drain (ICD) insertion. This can also be diagnosed with a CT scan or visualized through direct bronchoscopy.

Persistent air leak is defined when air flows freely back and forth between the bronchial tree and the pleural space. Air leak in the pleural space results in lung collapse, which compromises blood flow and gas exchange. The presence of an air leak can be detected by observing bubbling in the underwater seal chest drainage system, indicating the presence of a bronchopleural fistula (BPF). Bubbling may be consistently present or may gradually reduce over time. Additionally, when the patient coughs, bubbles may appear in the underwater seal, further confirming the presence of an air leak [13]. This characteristic bubbling is a valuable indicator to the presence of bronchopleural fistula.

Since air exists in the area where it should not be, physiological thoracic parameters get altered, opposing normal functions. Air leak will decrease the lung volume and airflow, hence increasing pleural pressure, resistance, and elastase. Tidal volume is dependent on pleural cavity. The decrease in lung volume results in decreased oxygenation and the accumulation of carbon dioxide. This brings up the "steal phenomenon," resulting in hypoxia, and is an adaptive process in the long term. The size of the air leak correlates proportionally to the degree of compromise. When air is released from the pleural cavity, a higher pressure is required to evacuate the pressure found in the pleural cavity. By releasing air from the pleural cavity, carbon dioxide gets washed out, decreasing the partial pressure of carbon dioxide and subsequently resulting in respiratory alkalosis [14].

Managing hydropneumothorax surgically involves several approaches, with the following two mainly used: video-assisted thoracoscopic surgery (VATS) procedure or open window thoracostomy. They both have advantages over one another, as VATS is favored in the presence of infected pleural fluids with bronchopleural fistula and holds fewer hospital stay days. Conversely, open window thoracostomy is the choice if empyema is present or anatomy with adhesions and scarring is a challenge [15].

With respiratory compromise, mechanical or functional, oxygenation is expected to be altered, acutely, chronically, or both. In relation to our patient, presenting acutely after the massive formation of mechanical obstruction, he has compensated to having normal or near-normal oxygenation. Cardiovascular and biochemical functions aid in the adaptation to hypoxia. In patients with no cardiac history, hypoxemia is tolerated given that the patient is not acidotic or has a decease in his mental state [16]. Hypoxia produces an increase in minute ventilation and cardiac output but not much effect on the biochemical level. On the contrary, at the biochemical level, hypoxia-inducible factor 1 (HIF-1) is the protein responsible to raise oxygen and to adapt to low oxygen in the blood [17]. It has been hypothesized that to achieve a better ventilation-perfusion match, lying on the large effusion side increases the hydrostatic pressure, which will further shift blood flow to the healthy lung [18].

## Conclusions

The activation of tuberculosis specifically in immunocompromised individuals results in unusual and possible lethal pulmonary sequelae. Hydropneumothorax is one of the rare complications that arise, which is further complicated with bronchopleural fistula. This necessitates the early recognition of BPF as this will define the treatment route, surgical or nonsurgical. Management is case-based and depends on the pathophysiology with the extent of involvement found. It is crucial to set a multidisciplinary approach to cover the multisystem involvement and provide optimum care.
